# School‐related worries in the day‐to‐day lives of early adolescent females: Links to positive affect and depressive symptoms

**DOI:** 10.1111/jora.70075

**Published:** 2025-09-22

**Authors:** Jennifer S. Silk, Kirsten P. McKone, Samantha R. Silk, Alexandra F. Petryczenko, Cecile D. Ladouceur

**Affiliations:** ^1^ Department of Psychology University of Pittsburgh Pittsburgh Pennsylvania USA; ^2^ Department of Psychology University of Minnesota Minneapolis Minnesota USA; ^3^ Department of Psychology Kenyon College Gambier Ohio USA; ^4^ Department of Psychology Penn State University University Park Pennsylvania USA; ^5^ Department of Psychiatry University of Pittsburgh Pittsburgh Pennsylvania USA

**Keywords:** academic, adolescence, depression, ecological momentary assessment, positive affect, school, stress, worry

## Abstract

There is currently a mental health epidemic among adolescents, with record high rates of depression, particularly among females. Although many potential causes of this epidemic have been suggested, very little attention has been paid to the school context, despite the fact that academic pressures have increased in recent decades. Intense pressure to excel is thought to play a role in the development of depression, but little is known about how worries about school might play a role in the mental health crisis. We used ecological momentary assessment (EMA) to assess day‐to‐day worries, including school‐related worries, in early adolescent females and the links between school‐related worries and daily affect, as well as depressive symptoms over 1 year. Participants were 117 youth assigned female at birth, ages 11–13 (*M*_age_ = 12.25[0.80]), with 2/3 at heightened risk for depression due to shy/fearful temperament. Across 16 days, youth reported a daily worry in the morning and rated their positive affect (PA) and negative affect (NA) 3–4 times per day. School‐related worries were the most frequently reported concerns, comprising 28% of total worries, more than triple the rate of any other categories. On days when school worries were reported, participants exhibited lower levels of same‐day PA, compared with when other types of worries were reported. There were no associations between school worries and daily NA. The intensity of school worries predicted higher depressive symptoms at 1‐year follow‐up, particularly for low‐risk participants. Findings suggest that school worries are pervasive among early adolescent females and may serve to dampen positive emotions, perhaps by diverting attention and time from potential positive experiences. School worries also contributed to increases in depressive symptoms over the course of 1 year, suggesting that school concerns warrant greater attention as a potential factor in the youth mental health crisis. Worries about school represent a potentially modifiable risk factor for depression, which could be addressed at the individual, family, school, and/or policy level.

## INTRODUCTION

There is currently a mental health epidemic among adolescents, especially females, with a 2021 CDC report revealing that 56% of female high school students reported significant depressive symptoms (Centers for Disease Control and Prevention, 2023). Little is known about the key factors driving this epidemic. One potential factor is concerns related to school. Academic pressures have increased in recent decades, with intense pressure to excel thought to play a role in the development of depression (Luthar et al., 2019; Steare et al., 2023). Yet, little is known about how academic worries may play a role in the mental health crisis. We used ecological momentary assessment (EMA) to assess day‐to‐day worries, including school‐related and other worries, in early adolescent females and the links between school‐related worries and daily affect and depressive symptoms.

### School worries in youth

Today's young people must grapple with a wide range of concerns, such as social media, climate change, global health challenges, gun violence, political unrest, discrimination on the basis of racial/ethnic, religious, or gender/sexual identity, and an intense pressure to excel, not to mention more typical domains of teen worries, such as peers, romantic relationships, and family (American Psychological Association, 2018; Fegert et al., 2020; Robert Wood Johnson Foundation, 2018). Yet, little is known about what concerns youth spend the most time worrying about (Owczarek et al., 2020), with worry defined as “a state of mental distress or agitation due to concern about an impending or anticipated event, threat, or danger” (VandenBos, 2007). Even less is known about how frequent and intense these worries are in the course of youths' daily lives, as most studies have used retrospective questionnaires. However, survey and qualitative data point to school worries as potentially the most pressing domain of worry for teens. For school worries, the anticipated event, threat, or danger is related to an academic, social, or extracurricular aspect of school, such as grades, tests, homework, teachers, presentations, or performance in after‐school activities. A narrative review of 12 qualitative and quantitative studies on adolescent worry found that school performance was the only domain of worry that had stable, high endorsement across studies (Owczarek et al., 2020). It should be noted though that only four studies focused on U.S. youth, all of which were conducted before 1995, thus research on the current domains of worry among U.S. adolescents is sorely needed. However, two more recent qualitative studies from the United Kingdom showed that school performance was a key area of concern (Fisher et al., 2017; Young et al., 2016).

It is likely that worries about school have increased in recent decades, following the rise of a widespread “achievement culture” that is predominant in many communities in the U.S. and around the world, which prioritizes excellence in academic and extracurricular endeavors as a necessary pathway for future success. This intense pressure to excel was a top concern expressed by U.S. researchers, advocates, funders, policymakers, and practitioners interviewed about adolescent well‐being through the Robert Wood Johnson Foundation (2018). This culture, which is especially prominent in high‐achieving schools (Luthar et al., 2019), likely engenders a high degree of worry about school‐related achievements in the classroom and in extracurricular spaces among adolescents across the spectrum of socioeconomic backgrounds and school types (Roderick et al., 2009). Although there has been no direct empirical research on how achievement culture affects youth's emotional adjustment, it has been demonstrated that youth in high‐achieving schools are at elevated risk for depression and other mental health problems (Luthar et al., 2019; Luthar & Kumar, 2018), which the authors interpreted as being related to intense pressure to excel. However, empirical research is needed to document how much youth are worrying about school on a day‐to‐day basis, how frequent and intense these worries are relative to other worries, whether effects are detectable in adolescents broadly (i.e., not limited to high socioeconomic status) and how they affect youths' emotional health, in both the short‐ and longer term.

### Associated constructs: Academic pressure and stress

Although there were a few early studies examining the content of teen worries that included school as an option, and school often arose as the most frequent category of worry in these studies (Owczarek et al., 2020), there has been no published research specifically examining school‐related worries in the modern era that we are aware of, despite increases in academic demands. Additionally, researchers have not examined the occurrence of school worries in the day‐to‐day lives of teens using ecologically valid methods. However, there has been some survey and retrospective research on the related constructs of academic pressure and academic stress that helped to inform our hypotheses, reviewed briefly here.

Academic pressure has been defined as the “fear of failure, concerns about the future, chronic stress about workload and exams, and worries about parental expectations and competition with peers for grades” (Steare et al., 2023, p. 2). Large‐scale survey research suggests that academic pressure is a common source of stress for young people around the world, for example, affecting 77% of U.K. adolescents and young adults in a large national survey (YoungMinds, 2019), and emerging as one of the top two problems endorsed across a large sample of Australian adolescents. In the U.S., a 2018 Pew Research Center survey revealed that pressure to do well in school was the most common source of pressure reported by teens, endorsed by 60% of respondents (Horowitz & Graf, 2019). Several studies have also shown that female students report higher levels of academic pressure than males (Fildes et al., 2014; Horowitz & Graf, 2019; Klinger et al., 2015). However, the construct of academic pressure is broad, with school worries constituting only one small component, therefore we aimed to focus more specifically on school worries, given the strong link between worry and depression (Vîslă et al., 2022).

Surveys have also examined the related construct of “academic stress,” defined as stress generated in the educational context by the demands of school life, such as excessive teachers' requirements and parental expectations, academic examinations, homework overload, and classroom competition (Barbayannis et al., 2022; Jiang et al., 2021). For example, a 2020 survey of 540,000 students across 72 countries found that 66% of students reported feeling stressed about grades and 55% of students felt stressed about tests (Pascoe et al., 2020). Girls reported higher levels of stress related to schoolwork compared with boys. Worry and stress are bidirectionally related, with worry conceptualized as both a trigger for and response to stress (Brosschot et al., 2006). Therefore, more research is needed to better understand what types of specific worries may be underlying and sustaining these high levels of academic stress in youth.

Finally, there is also some evidence that academic pressure is increasing, consistent with the aforementioned concerns about achievement culture. Data from 32 countries in Europe and North America revealed that perceptions of academic pressure increased from 2002 to 2018, but only for girls (Löfstedt et al., 2020), highlighting the importance of understanding the impact of factors that contribute to academic stress, such as school worries, on emotional health, especially in girls.

### School‐related worries and adolescent depression

One aspect of emotional health that school worries are likely to impact is the development of depression. Rates of depression are alarmingly high among adolescents, with a dramatic spike in recent decades, especially among girls (Centers for Disease Control and Prevention, 2023). Meta‐analytic work shows that worry is a well‐established risk factor for later depression (Vîslă et al., 2022). School‐related worries might impact the development of depression through altering youths' day‐to‐day emotional experience, as worry has been shown to increase negative affect (NA) and decrease positive affect (PA) in young people (McLaughlin et al., 2007). School‐related worries might also prevent youth from engaging in or enjoying rewarding activities, either because they feel they need to spend all their time on schoolwork, or because the worry‐related cognitions compete for attention with more positive thoughts (Silk et al., 2012), potentially contributing to anhedonia.

Few studies have focused specifically on school‐related worries, but researchers have shown that the broader constructs of academic pressure and academic stress are associated with depressive symptoms in adolescents. A meta‐analysis of studies conducted up to 2022 showed a consistent positive association between academic pressure and stress with depressive symptoms in children and adolescents (Steare et al., 2023). However, most studies were cross‐sectional and utilized retrospective reports, often with a single item, limiting directional inference and a more nuanced understanding of the types of academic pressures affecting youth. Of the cross‐sectional studies, all reported a positive association between academic pressure/stress and depressive symptoms. However, results from the three longitudinal studies included in the meta‐analyses were more mixed. Kaman et al. (2021) found that among German adolescents, academic pressure was positively associated with depressive symptoms longitudinally, but only among boys. Fu et al. (2022) found that academic stress at baseline was positively associated with depressive symptoms 1 year later using a large sample of youth in the China Education Panel survey. In contrast, in a study of 520 English adolescents, there was no evidence that depressive symptoms increased as academic stress increased, such as during examination periods (Locker & Cropley, 2004). Furthermore, there is little research examining links between academic pressure/stress and depressive symptoms among U.S. adolescents, despite reports that perceived academic pressure is higher among U.S. youth compared with other European and North American countries (Klinger et al., 2015). Finally, as described above, while the broad constructs of academic pressure and stress are related to worry, more nuanced research is needed to understand how worry about school may play a more specific role in the development of depression.

### Within‐person effects on day‐to‐day emotion

There is also very little known about how school‐related worries might impact day‐to‐day emotional health in the shorter term, such as same‐day PA and NA. Establishing these links has important implications for adolescent mental health, as alterations in daily emotional experience in adolescence – specifically higher levels of NA and lower levels of PA – are associated with increased risk for depression and other affective disorders in children and adolescents (Houben et al., 2015; Reitsema et al., 2022). Identifying specific drivers of increases or decreases in these emotions in real time could suggest modifiable targets for improving emotional health and preventing depression and other mental health problems in youth.

There are a number of mechanisms through which school worries might affect daily emotions. School worries might engender higher levels of NA throughout the day, spreading into other domains and experiences of daily life. As discussed above with regard to depression, it may also be that school worries cause a narrowing of attention toward associated negative thoughts (Fredrickson & Branigan, 2005), pulling cognitive and attentional resources away from positive thoughts and experiences, resulting in reduced levels of PA in daily life as well. One study investigated within‐person changes in affect during the days surrounding an examination, finding that youth indeed reported reduced PA and increased NA before a mid‐term exam (Sang et al., 2018). But it is not known how more typical day‐to‐day school worries might influence emotional experience.

One method that may shed additional insight on the near‐term effects of school worries among youth is EMA, a method that utilizes signaling devices, such as smartphones, to sample participants' thoughts, emotions, and behaviors in real time. Mengelkoch et al. (2023) recently highlighted the potential utility for EMA to move stress research beyond the limitations of retrospective self‐report to reveal dynamic associations between real‐time stressors and subsequent psychological states at the micro‐level. In the only EMA study, we are aware of to examine academic stress, there were no within‐person associations between academic stress and same‐day NA or PA (Xu et al., 2023); however, academic stress was defined in a relatively limited way (“did not understand something taught in class,” “did poorly on a test, quiz, or homework,” or “did not turn in homework that was due”). In the present study, we used participants' own idiographic self‐reported worries about school‐related issues as predictors of fluctuations in PA and NA across days.

### Potential moderators of the link between school worries and depressive symptoms

It is also likely that school‐related worries may affect youth differentially depending on individual differences in intrapersonal traits or demographic characteristics. For example, youth who are high in shyness/fearfulness are known to be at elevated risk for developing depression (Karevold et al., 2009, 2012; Murberg, 2009). According to the well‐validated diathesis‐stress model of depression (Monroe & Simons, 1991), intrapersonal characteristics such as a shy/fearful temperament may serve as a diathesis that predisposes youth toward depression when activated by a stressor. Although research to date has not explored academic stress within this framework, it is likely that academic stress could exacerbate pre‐existing vulnerabilities toward depression, such as a temperamental style marked by shyness and fear. Additionally, there is evidence that academic pressure may be particularly detrimental within the context of high‐achieving schools (Luthar et al., 2019); therefore, there is a need to understand whether socioeconomic status might moderate the link between academic worries and the development of depressive symptoms.

### Current study

To address these gaps in the literature, we used EMA as an ecologically valid approach to assess daily worries in a sample of early adolescents identified female at birth that was enriched for temperamental risk for depression. We focused on youth identified female at birth given higher rates of both depression (Centers for Disease Control and Prevention, 2023; Hankin et al., 2015) and academic stress/pressure (Fildes et al., 2014; Horowitz & Graf, 2019; Vansoeterstede et al., 2023) in this population. We focused on early adolescence as a time when school demands begin to increase, worry becomes more prevalent (Kertz & Woodruff‐Borden, 2011), and depressive symptoms begin to rise, especially for girls (Ge et al., 2001). The sample was enriched for shy/fearful temperament in order to increase variability in the development of depressive symptoms. First, we hypothesized that school worries would be more common than other types of daily worries, such as worries about peers, family, health, and social media/digital technology. Second, at the within‐person level, we hypothesized that on days when participants reported a school worry, they would also report higher levels of NA and lower levels of PA, compared with days when they reported other types of worries. Third, at the between‐person level, we hypothesized that more frequent and more intense school worries across the 16‐day EMA period would be associated with higher levels of depressive symptoms at 1‐year follow‐up, especially for female adolescents at high temperamental risk for depression and from higher Socioeconomic status families.

## METHOD

### Participants

Participants for the current report were 117 youth identified female at birth (based on parent report) ages 11–13 participating in a longitudinal study designed to examine emotional, social, and neural factors in the development of social anxiety and depression. It was designed such that approximately two‐thirds of participants would be at elevated risk for depression based on elevated dispositional shyness/fearfulness, which was operationalized as scores 0.75 *SD* higher than the mean on the Fear and/or Shyness subscales of the Early Adolescent Temperament Questionnaire‐Revised (EATQ‐R; Ellis & Rothbart, 2001). The remaining participants were at typical risk. Participants were recruited from community advertisements, online advertisements, a university research registry, and referrals from pediatricians and other research studies in a large Midwestern city. The full sample comprised 129 adolescent participants, with usable EMA data available for the 117 included in this report. Gender identity was not assessed. As shown in Table 1, the sample was 68% White, 19% Black, 9% Multiracial, and 4% other racial identity, matching the demographics of the local area. Additionally, 9% identified as Latine or Hispanic. Families reported a wide range of socioeconomic status, with annual family income ranging from less than $20,000 to greater than $100,000, with a median of $80,000 to $90,000. The majority of participants attended public school (76.9%).

**TABLE 1:** Participant (*N* = 117) demographics and clinical information.

	*M*	SD
Age	12.25	0.80
Race/Ethnicity	***N***	**%**
Asian/Asian American	2	1.7
Biracial	11	9.4
Black/African American	22	18.8
Native American	1	0.9
Other racial background	1	0.9
White/European American	80	68.4
Latine or Hispanic	10	8.5
Total Family Income (Annual)		
$0–20,000	10	8.7
$20,001–40,000	9	7.8
$40,001–60,000	16	13.9
$60,001–80,000	18	15.7
$80,001–100,000	16	13.9
$100,001+	46	40.0
School Type		
Public	90	76.9
Private	18	15.4
Home or CyberSchool	9	7.6
Diagnoses		
Specific phobia	18	15.4
Attention‐deficit/hyperactivity disorder	6	6.1
Oppositional defiant disorder	5	4.3
Unspecified disruptive behavior disorder	1	0.9
Adjustment disorder	1	0.9
Enuresis	1	0.9
Tic Disorder	2	1

Diagnostic exclusion criteria included current diagnosis or history of any DSM‐5 anxiety disorder (except specific phobia), major depressive disorder, autism spectrum disorder, bipolar disorder, or schizophrenia, as determined by the Kiddie‐Schedule for Affective Disorders and Schizophrenia (Kaufman et al., 2013). Given the study's focus on predictors of the development of depressive symptoms, we excluded current or past internalizing disorders so that results could be interpreted in relation to the development of these disorders. Specific phobia was allowed because it tends to have a narrower scope and lower functional impairment. We also excluded severe psychopathology (autism spectrum disorder, bipolar disorder, and schizophrenia), which are associated with extreme variation in emotional experience (American Psychological Association, 1994). Additional exclusion criteria included IQ < 80 (assessed with the Wechsler Abbreviated Scale of Intelligence; Wechsler, 1999), current or past neurological or serious medical conditions, fMRI contraindications, uncorrected impaired vision, past head injury or neurological anomalies, and current use of medication that impacts the central nervous system. Diagnostic data for enrolled participants are shown in Table 1.

### Procedure

All procedures for the study from which these data were drawn were approved by the University's Institutional Review Board. At a baseline visit, participating youth and their parents provided informed assent and consent, respectively. Following informed consent, the WASI (Wechsler, 1999) and the K‐SADS‐PL (Kaufman et al., 2013) were administered to determine eligibility, and teens and parents completed questionnaires. At a second laboratory visit, approximately 2 weeks following the initial visit, adolescents and their parents completed a number of observational tasks not pertinent to the present study. At the end of this visit, participants were given a pre‐programmed Android smartphone and instructed on how to complete the 16‐day EMA protocol using the Web Data Express app. The EMA protocol was comprised of questions related to adolescents' social context, emotions, thoughts, and activities, and began the Saturday following the participant's second lab visit and continued for the following 16 days (six weekend days and ten weekdays). (Of note, although some participants had cellphones at the time of the first wave of the study [2016–2018], many did not, and the high average compliance rate of over 80% suggests that carrying a second phone was not overly burdensome.) Finally, participants were sent online questionnaires at the 1‐ year follow‐up to assess their depressive symptoms.

### EMA

During the EMA portion of the study, adolescents received a notification to complete a survey three times per day on weekdays (once in the morning between 7 AM and 8 AM and twice in the afternoon and evening) and four times per day on the weekends (blocks: 11–1:30 1:30–4:00, 4:00–6:30, 6:30–9:30), for a maximum of 54 observations. Prompts were sent randomly within the aforementioned time blocks. Surveys expired after 1 h. Participants completed an average of 81.3% of prompts (SD = 13.9%, range = 37.0%–100%). At the second lab visit, the participant reported their typical wake time, which was used to anchor the timing of the first prompt. On weekdays, the remaining two prompts were delivered randomly within two blocks (one prompt per block; 3:30–6:30 and 6:30–9:30). Prompts were not issued during school hours to minimize disruption to learning and limit bias related to varying school policies restricting phone use.

### Measures

#### Depressive symptoms

At baseline and 1‐year follow‐up, participants completed online questionnaires via Qualtrics assessing their depressive symptoms using the 33‐item Mood and Feelings Questionnaire (Angold et al., 1995). The MFQ, which is well‐validated in child and adolescent samples (Daviss et al., 2006), requires participants to rate their depressive symptoms over the past 2 weeks (e.g., “I felt miserable or unhappy”; “I blamed myself for things that weren't my fault.”) on a scale of 0–2, where 0 = “Not True” and 2 = “True.” Researchers have found that a score of 29 best distinguished adolescents with depression from those without (Daviss et al., 2006). Scores were summed to create a total score. Reliability was high at both baseline (ɑ = 0.89) and 1‐year follow‐up (ɑ = 0.95).

#### Risk status

As noted above in the Participants section, the study was designed such that approximately two‐thirds of participants would be at elevated risk for depression based on elevated dispositional shyness/fearfulness. Risk status was operationalized as follows: Participants who scored 0.75 *SD* higher than the mean on the Fear and/or Shyness subscales of the Early Adolescent Temperament Questionnaire‐Revised (EATQ‐R; Ellis & Rothbart, 2001) were considered at elevated risk. The remaining participants were at typical risk. A binary variable was coded such that 0 = “typical risk” and 1 = “elevated risk.”

#### Demographics

Adolescent age at the baseline visit was calculated in years. Socioeconomic status was approximated by asking parents to report annual gross family income in dollars on a 0–10 scale, where 0 = 0–10,000 and 10 = 100,000+.

#### Daily worries

The EMA protocol included a daily morning question asking, “What are you most worried is going to happen today?” with an open‐text response field where the participants could type the contents of the worry. They were also asked to rate the intensity of their worry on a 0–100 scale (0 = “Not at all,” 100 = “Extremely,”) with the slider anchored at 0. Two coders classified responses into 13 categories (98% agreement). Categories were determined via consensus between the 2 coders and the lead author based on a review of the data and a preliminary round of coding, and covered 96% of responses, suggesting that they captured the majority of naturally occurring daily worries among our sample. Categories included: nothing, school (e.g., worried about school work, teachers, or activities), health (e.g. worried about one's own health or the health of others), family (e.g., worried about family members), peer (e.g., worried about issues with others in one's own age range), inconvenience (e.g. concerns about being late, losing something, or not getting a preferred food or material item), boredom or doing something aversive (e.g. worried they will be bored or have to do chores), logistics (e.g. worried they will not be able to go to a desired event, will forget something, or not know how to do a task), weather (e.g. worried it will rain), appearance (e.g. worried about hair, clothes, or body appearance), and social media/technology (e.g. worried about something on social media apps, other online activities and interactions, or video games). An “other” category was included for all other worries. Given the focus of this study, for descriptive purposes, school worries were further coded into subcategories, including educational (i.e., related to classwork, tests, homework), extracurricular, social‐peer (i.e., worries about social interactions with peers explicitly occurring at school, such as a friend being absent or embarrassing themselves in front of a peer in choir or a class presentation), and social‐nonpeer (i.e., worries about social interactions with teachers or other adults explicitly occurring at school), as well as other school worries that did not fit into any of the above categories. A binary indicator variable was created to indicate whether a school‐based worry was reported at each occasion and used in momentary analyses as the within‐person component. Person‐specific proportion scores of the binary indicator were calculated to serve as the between‐person component.

#### Daily affect

At each prompt, participants were asked to rate how they were feeling at the moment they received the prompt. They rated their affect on four positive (happy, joyful, excited, interested) and four negative (sad, mad, worried, stressed) emotions. Affect was rated using a sliding scale from 0 to 100 (0 = “Not at all,” 100 = “Extremely”), with the slider initially anchored at 0. Mean daily PA and NA were calculated by first identifying the maximum emotion reported at a given prompt and taking the means of the maximum across each day's timepoints within‐valence. Reliability was adequate (PA [*α*_within_ = 0.80, *α*_between_ = 0.94]; NA [*α*_within_ = 0.67, *α*_between_ = 0.94]).

### Analytic plan

First, descriptive analyses were conducted in SPSS (Version 28) to examine the frequency of worries reported. Second, given that worries were repeated within subjects, a repeated measures ANOVA was used to compare the frequency of school worries to other worry categories. In order to represent the major categories of worries without including an inordinate number of categories in the model that would reduce statistical power, we made the decision to include all categories of worry that were reported at least 5% of the time across the 16‐day EMA period. To examine the within‐person association between daily worries and affect, we leveraged multilevel models for intensive longitudinal data, which accommodates the nesting of observations within persons and allows for effects to be decomposed into between‐ and within‐person levels. Means‐only models indicated that there was sufficient variability at both the between‐ and within‐person levels. Intraclass correlations indicated that 57% of the variance in NA was at the between‐person level, whereas 66% of the variance in PA was at the between‐person level. Day‐level PA and NA were modeled as the outcome. To model within‐person effects of school‐related worries on daily affect, a binary variable was included, in which a value of ‘1’ indicated that the participant reported their primary worry for the day was school‐related, and ‘0’ indicated the participant reported a worry in another category (e.g., friends, family). To model the between‐person effects of school‐related worries on daily affect, proportion scores were calculated that reflected the overall percentage of days in which a given participant reported a school‐related worry out of the total days on which a worry was reported.

Finally, linear regressions were conducted to examine the associations between adolescents' frequency of reporting school‐related worries (i.e., the between‐person proportion of days on which participants reported a school‐related worry out of the total days worries were reported), the intensity of those worries (i.e., person‐means of daily intensity), and depressive symptoms, controlling for age and baseline (T1) depressive symptoms. Moderation analyses were conducted to evaluate whether the association between school worries and depressive symptoms was dependent on temperamental risk for depression and/or socioeconomic status.

## RESULTS

### Frequency and intensity of worries

Table 2 presents the breakdown of participants' worries and the number of participants who reported each type of worry. School worries represented the largest share of the worries, making up 28.14% of reported worries, and occurred on 28% of days. Most school worries were related to educational aspects of school (63.47% of school worries), with less frequent reports of worries related to extracurricular activities and social worries related to school. The next most frequent category of worries was “nothing” (i.e., no worries), reported on 23.15% of worry responses. All other categories of worries were reported on less than 9% of responses, including health (8.61%), family (6.19%), peers (5.99%), and social media/technology (1.02%). Only 3.76% of worries were about topics not included in our coding scheme.

**TABLE 2:** Frequency of worries reported at T1.

Worry category	% Days worry reported	% participants Who reported worry
School	28.14	64
Educational	17.86	53
Extracurricular	3.64	24
Social (peer)	3.13	22
Social (nonpeer)	0.77	9
Other	2.74	19
Nothing	23.15	62
Logistics	8.99	53
Health	8.61	45
Family	6.19	44
Peer	5.99	39
Inconvenience	5.48	37
Bored/Aversive	4.97	32
Other	3.76	32
Weather	2.61	16
Appearance	1.08	13
Social Media/Technology	1.02	9

The repeated measures ANOVA confirmed that as hypothesized, school worries were significantly more frequent than any other category (*F*(3, 384) = 23.29, *p* < .001), with post hoc pairwise comparisons indicating that participants worried significantly more about school (*M* = 3.28, SE = 0.32) compared with each of the other worry categories (all *p*s < .001).^1^ In terms of the number of participants reporting each category of worry, school‐related worries were again the most common, endorsed at some point by 64% of participants. Other worries reported by one‐third to one‐half of the sample included logistics (53%), health (45%), family (44%), peers (39%), and inconvenience (37%). There was no difference in the average intensity of school‐related worries (*M* = 44.86, *SD* = 24.43) and nonschool‐related worries (*M* = 43.13, *SD* = 22.38; *t* = 0.65, *p* = .518).

### Daily associations between school worries and affect

Preliminary analyses revealed that levels of daily NA were relatively low (*M* = 18.10, *SD* = 21.18, range = 0–100), while levels of daily PA were higher (*M* = 67.87, *SD* = 23.92, range = 0–100). Multilevel models indicated that school worries were associated with less daily PA at the within‐person level, such that, adjusting for a participant's average tendency to worry about school, on days when her primary worry was on a school‐related topic, she reported lower levels of PA for that day than on days when she reported a different primary worry (e.g., family, friends; *B* = −3.01, *p* = .004). Additionally, we conducted sensitivity analyses to ensure the results did not change when worries related to extracurricular activities were removed from the “school worries” category, or when covariates (family income, race, school type) were included in the model. Results did not change. School worries were not associated with same‐day NA (*B* = 0.05; *p* = .41). Results were replicated with no changes in significance when substituting the average of all four positive and negative emotions (i.e., daily mean of momentary means) for the average of the maximum positive/negative emotion at each timepoint (i.e., daily mean of momentary maximum).

### Associations between school worries and depressive symptoms

**FIGURE 1:**
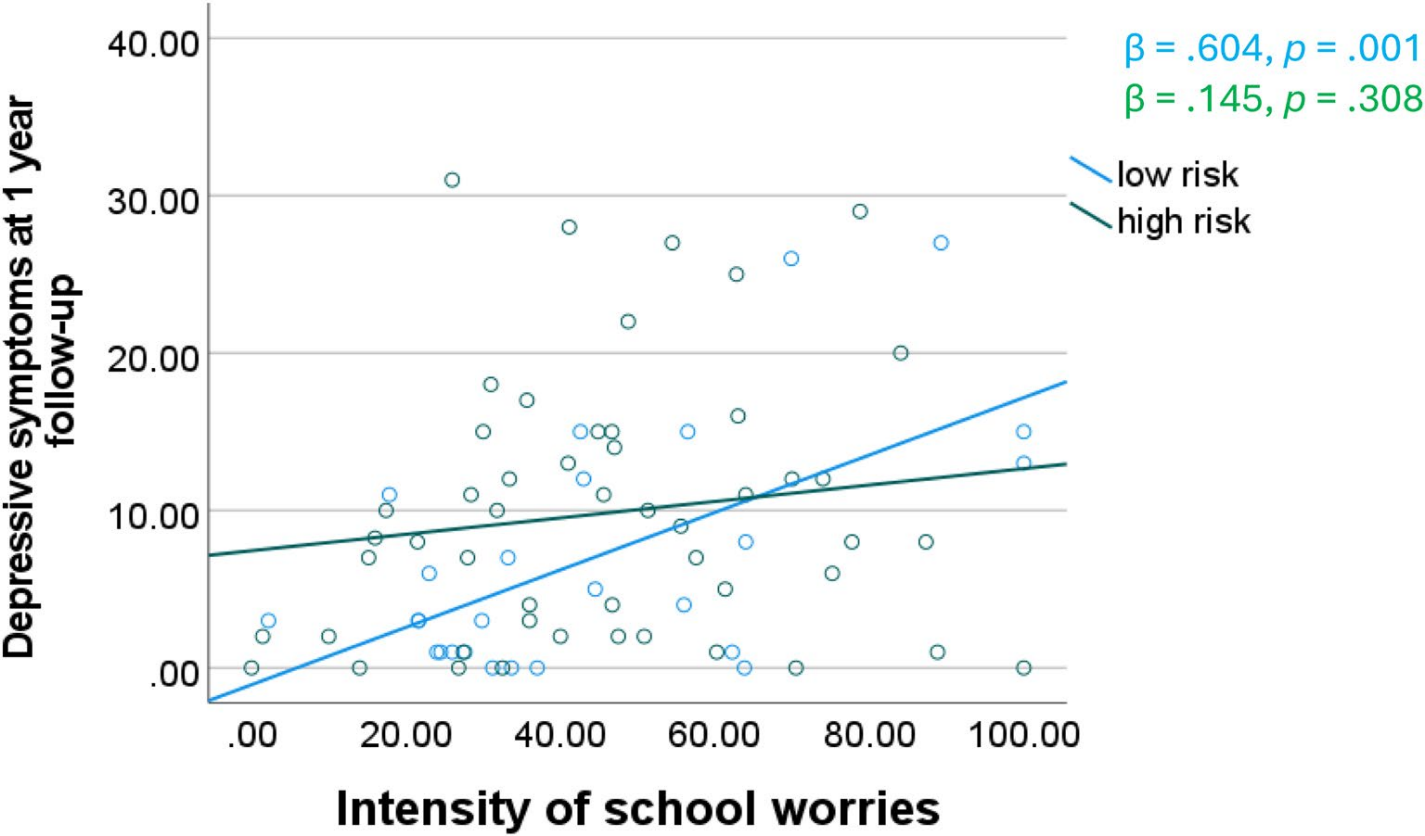
Intensity of school worries predicts depressive symptoms 1 year later for low‐risk participants, controlling for age and baseline depressive symptoms.

Contrary to hypotheses, there was no association between the frequency of school worries and 1‐year follow‐up depressive symptoms (*B* = .07, *t* = 0.21, *p* = .835), nor any interactions with risk status (*B* = 0.01, *t* = 0.02, *p* = .982), or SES (*B* = .10, *t* = 0.45, *p* = .656). However, in the subsample of participants that reported a school worry (*N* = 79), the intensity of school worries predicted depressive symptoms 1 year later, controlling for age and baseline depressive symptoms (*B* = .80, *t* = 2.23, *p* = .029). Furthermore, the intensity of school worries interacted with risk status (*B* = −.89, *t* = −2.15, *p* = .035) such that, as shown in Figure 1, more intense school worries predicted increases in depressive symptoms for low‐risk, but not high‐risk participants. Intensity of school worries did not interact with SES to predict 1‐year depressive symptoms (*B* = .23, *t* = 0.71, *p* = .478).

## DISCUSSION

This real‐time investigation of daily worries shows that school‐related worries are the most common daily concerns reported by 11 to 13‐year‐old female adolescents, far surpassing worries about family, health, friends, social media, and other concerns. These worries were associated at the day level with decreased PA, suggesting that worries about school are associated with dampened mood and thus may interfere with the ability to enjoy other experiences. Typical youth with more intense school worries were at higher risk for developing depressive symptoms over a year, although temperamentally shy/fearful youth were less impacted by school worries, perhaps being more impacted by social issues. Findings suggest that pervasive worry about school warrants greater attention as a potential factor in the youth mental health epidemic.

We used EMA to classify the content of idiographic worries among adolescents in their daily life, improving the ecological validity of previous research. We found that when participants were asked to report prospectively on their biggest worry for the upcoming day, school‐related worries topped the list. These worries were reported by the majority of participants (64%) and occurred on 28% of days, which was more than triple the frequency of all other worry categories (ranging from 1% to 9% of days). The majority of these worries were related to educational aspects of school, such as tests, projects, and homework, with much fewer worries about extracurricular and social aspects of school, although we did not have sufficient power to examine differential effects of different school worry subtypes. These findings are consistent with retrospective survey‐based research showing high rates of academic pressure and stress among adolescents (Pascoe et al., 2020; Vansoeterstede et al., 2024; YoungMinds, 2019), especially girls (Fildes et al., 2014; Giota & Gustafsson, 2017; Klinger et al., 2015; Löfstedt et al., 2020), but extend the findings to the more specific and targetable construct of worry. The results also improve the ecological validity of previous research and extend findings to earlier in adolescence. Collectively, these findings show that worries about school are pervasive among early adolescent females and warrant further attention for their role in mental health and well‐being.

It was interesting to note that the youth in our study were somewhat less worried than might be expected about topics that get far more attention in the literature, such as peers, family, and social media. Although popular media and some researchers (e.g. Haidt, 2024; Twenge et al., 2017) have portrayed smartphone technology and social media as primary drivers of the youth mental health crisis, only 11 participants (9%) ever reported a worry related to social media or technology, which occurred on only 1% of days across the sample. Similarly, although peer rejection is a known contributor to mental health challenges among youth (Rubin et al., 2009), the adolescents in our study were not very worried about their peer relationships on a day‐to‐day basis, with only 39% of participants ever reporting a peer‐related worry, which occurred on 6% of days across the sample. This is particularly surprising since our sample was enriched for shy and fearful temperament, a population which tends to be more sensitive to peer rejection (Jarcho et al., 2019). Family worries occurred at a similar rate to peers, affecting 44% of participants and also occurring on about 6% of days, suggesting that worries about family life were not pervasive.

The most common category of worries following school was actually “logistics” occurring on 9% of days and for 53% of participants. This suggests that much of youths' worries were about practical aspects of their day, like whether they would be able to attend a desired event or be able to figure out how to accomplish a task. Health concerns also occurred on close to 9% of days (45% of participants), but for the most part reflected more minor health concerns like stomachaches, headaches, having a cold, or feeling tired. Other categories that emerged from the data reflected more minor aspects of daily life, such as weather, inconvenience, boredom, having to do something they did not want to do, and appearance. This suggests that the adolescents in our study were more worried about the minutia of day‐to‐day life than their relationships with family and friends. Additionally, only 3.76% of days included worries outside those captured in our coding system, meaning that youth rarely reported worries about larger societal or global issues, such as politics, climate change, or discrimination. The temporal nature of the survey question, which explicitly asked about worries for the upcoming day, may be one reason why these broader worries rarely occurred.

We also examined real‐time links between school‐related worries and day‐to‐day fluctuations in PA and NA. Surprisingly, we did not find any associations between school‐related worries and same‐day NA. This may have been the result of a floor effect, given that NA ratings were typically low (*M* = 18 out of 100). It is possible that NA may have been higher in a clinical (as opposed to high risk) sample, providing more power to detect this association. It is also possible that school worry has a stronger effect on day‐to‐day PA compared with NA, given that we found that school worries were related to the daily experience of positive emotion. Specifically, as hypothesized, we found that PA was lower than usual on days when participants reported a school‐related worry. This is consistent with one previous study that found lower levels of PA in youth before a mid‐term exam (Sang et al., 2018), but extends the finding to more typical day‐to‐day school concerns.

Our findings suggest that school‐related worries dampen PA throughout the day. This might occur through several potential mechanisms. First, when youth are worried about their school performance, they might spend so much time studying and doing homework that they are unable to engage in other rewarding and PA‐generating activities. This is an important possibility, as a recent survey of more than 50,000 high school students, mostly from high‐achieving schools, showed that students reported doing an average of 2.7 h of homework per night (Pope, 2018). Second, school‐related worries may narrow youth's attention toward these more negative thoughts (Fredrickson & Branigan, 2005), pulling cognitive and attentional resources away from positive thoughts and experiences. Future research is needed to better understand how these and other mechanisms might link daily worries about school to dampened PA. Although the precise mechanisms are unclear, the finding that school worries are associated with reduced PA has important implications given the critical role that PA plays in well‐being among youth (Reitsema et al., 2022). In fact, as described in the “broaden and build” theory of positive emotions, PA serves to broaden attention and increase the flexibility of behavioral response repertoires (Fredrickson & Branigan, 2005; Fredrickson & Joiner, 2002). PA also predicts stronger student engagement in school, therefore reduced PA could adversely affect school engagement and performance (Reschly et al., 2008). Finally, lower levels of daily PA may contribute to the development of anhedonia, a key symptom of depression.

We also found that, among the typical youth in our study who did not demonstrate a shy and fearful temperament, more intense school‐related worries were associated with higher rates of depressive symptoms over the course of a year. This replicates a large body of survey‐based cross‐sectional research showing that academic stress or academic burden is related to depressive symptoms (Steare et al., 2023) but extends this literature in several ways. First, using a more ecologically valid and personalized measure of school‐related worries, compared with the broad constructs of stress and pressure, we found support for a longitudinal link with depression, which has been inconsistent in some previous research (Fu et al., 2022; Kaman et al., 2021; Locker & Cropley, 2004). Second, we found that the intensity of worries about school was more important than the frequency of these worries in understanding the development of depressive symptoms, which has important methodological implications for assessing school concerns. Third, we found that the link between school‐related worries and depressive symptoms differed for youth with and without a shy/fearful temperament. The fact that school‐related worries were differentially related to mental health outcomes based on temperament might help explain inconsistent findings in previous longitudinal studies (Fu et al., 2022; Kaman et al., 2021; Locker & Cropley, 2004). While we expected this link to be stronger among the shy/fearful participants, we actually found that the link between school‐related worries and depressive symptoms was attenuated among shy/fearful female adolescents, although these analyses may have been underpowered, suggesting a need for replication. It may be that for shy and fearful youth, social worries play a more important role in the development of depressive symptoms than school‐related worries. For example, although they may report a school worry in the morning, social concerns that occur throughout the day may supplant school worries and have a greater impact on their functioning. Future research is needed to disentangle the different ways in which school‐related worries may affect youth with different temperaments. However, the present results suggest that for youth who are not socially reticent, school‐related worries may be an important factor in the development of depression from early to mid‐adolescence. Additionally, although our study focused only on depressive symptoms, worries about school might also serve as a transdiagnostic risk factor for other forms of internalizing psychopathology. For example, academic stress has also been linked to anxiety, nonsuicidal self‐injury, and suicidality (Chen et al., 2024; Steare et al., 2023) with evidence that depressive symptoms may mediate the link between academic stress and suicidal ideation (Ang & Huan, 2006).

Future research is needed to understand the mechanisms through which school worries contribute to increases in depressive symptoms. Our results suggest that the dampening of PA, which is a key feature of depression, may be one important avenue to explore. Other mechanisms through which school worries might influence depressive symptoms include impacts on self‐esteem (Giota & Gustafsson, 2017) and interpersonal relationships (Lan et al., 2023). For example, when youth are worried about school, they may spend more time focusing on their schoolwork, allowing less time for social connection, eventually taking a toll on their sense of belonging and contributing to risk for depression. When adolescents are worried about school, this may also interfere with their sleep (Liu et al., 2021). Since sleep problems are a key risk factor for the development of depression in youth (Gregory et al., 2005), disturbed sleep may be another mechanism worth investigating.

The present study focused on early adolescent females, ages 11–13, who were typically in middle school. Middle school represents a time when academic demands begin to increase and rates of depression begin to rise, making early adolescents a key population on which to focus this research. However, academic pressure is likely to become more pervasive as youth progress through high school and pressure for college admissions becomes more intense, potentially increasing the frequency and intensity of school worries. Thus, it will also be important to extend this work to older adolescents and potentially even emerging adults, given that there is evidence of high levels of academic stress among college students (Barbayannis et al., 2022). Additionally, our study focused only on youth identified as female at birth. This is important, as there is clear evidence that females take school more seriously and spend more time on their schoolwork compared with males (Houtte, 2004; Trautwein & Lüdtke, 2009), and are at higher risk for depression (Centers for Disease Control and Prevention, 2023). However, it will be important to conduct research examining how school‐related worries affect day‐to‐day functioning and longer term depressive symptoms among youth identified as male, as well as youth with varying gender identities.

School concerns represent potentially modifiable risk factors for mental health, which could be addressed at the individual, family, school, and/or policy level (Steare et al., 2023). The results of our study underscore concerns that the achievement culture predominant in the U.S. (Luthar & Kumar, 2018; Robert Wood Johnson Foundation, 2018), as well as many other cultures worldwide (Jiang et al., 2021; Löfstedt et al., 2020), may have detrimental effects on the day‐to‐day well‐being and longer term mental health of youth, especially girls. The fact that school was the predominant source of worry for our participants, and that these worries were associated with reduced day‐to‐day positive well‐being, may suggest that too much pressure is being placed on youth to excel in their academic endeavors. Additionally, qualitative review of the open‐ended worry prompts indicated that many youth reported worries about not being able to finish the amount of homework and schoolwork assigned to them, consistent with some concerns that the quantity of homework in many schools is excessive (Guo et al., 2024). There is debate about the pros and cons of homework among education professionals (Zuzanek, 2009), with some evidence that too much homework may even have adverse effects on achievement (Guo et al., 2024; Shao et al., 2024). Therefore, schools may benefit from exploring the extent to which their homework practices are having positive outcomes versus causing excessive worry in students, and modify such practices accordingly. Schools could also help students learn to better plan out their assignments so that they can be accomplished more efficiently, such as by managing social media distractions.

School systems could also provide better access to mental health prevention and intervention programs designed to help youth cope with school worries (Giota & Gustafsson, 2017; Pascoe et al., 2020). Families also play an important role in conveying school pressure (Haspolat & Yalçın, 2023) and research suggests that parental support predicts improvements in coping with school stress over the course of a year (Zimmer‐Gembeck et al., 2023). Therefore, it may be important to incorporate parents into prevention and intervention programs to help them learn to reduce academic pressure on youth and increase support, in turn reducing youths' school‐related worries.

It is important to consider the present findings in consideration of several limitations. As previously mentioned, the results are limited to adolescents identified female at birth, many of whom were at temperamental risk for depression, and the majority of whom were White and in public school. While the at‐risk sample and focus on females was a strength for studying risk for depression, it nevertheless limits the extent to which findings might extend to samples not selected based on temperament, youth identified male at birth, and gender diverse youth. It is also unclear how findings might generalize to youth from different racial/ethnic backgrounds and school settings. We excluded previous internalizing disorders in order to be able to examine predictors of the development of internalizing symptoms, specifically depressive symptoms, but included specific phobia given its more limited impairment (Stinson et al. 2007). Additionally, we excluded disorders that are associated with atypical daily emotional experience, such as schizophrenia, bipolar disorder, and autism spectrum disorder (American Psychological Association, 1994), as well as youth with ADHD with hyperactivity, due to a neuroimaging component unrelated to the present analysis. These inclusion and exclusion criteria render the sample ideal for our overarching goal of examining day‐to‐day emotional functioning and the unique emergence of depressive symptoms, but reduce the representativeness of the sample. Additionally, the sample represented a range of socioeconomic backgrounds. This heterogeneity in SES made it difficult to detect effects that might be specific to youth in high or low SES families. For example, although SES did not moderate the link between school worries and depression in the present sample, future research conducted with a larger sample of participants from high SES backgrounds and/or high‐achieving schools might reveal greater impacts of school worries in this population. Additionally, school concerns might be especially pronounced and detrimental among ethnic/racial minority youth in high‐achieving schools (Luthar et al., 2019), which we were not able to explore.

Our method of assessing school worries using an open‐ended question at the beginning of each day, while largely a strength, also introduced some limitations, in that it focused youth on worries occurring within a specific timeframe (i.e., that day), rather than potentially more diffuse and elusive but equally important concerns. Additionally, because the vast majority of school worries focused on academic aspects (e.g., tests, homework), we were not able to examine potential differential effects of other types of school worries (e.g., extracurricular activities). Some of the categories were also very broad, such as “logistics” and could benefit from being broken down further in a sample with more instances of these events. We also did not have information about whether days were “typical” or occurred during exam or testing periods, which might contribute to different levels of affect and stress. Finally, our between‐person moderation analyses examining the association between academic worries, risk status/SES, and depressive symptoms required constraining the sample to a subsample of *n* = 79 participants who had reported an academic worry at least once, which likely resulted in those analyses being underpowered to detect significant effects, although a significant effect did emerge. Despite these limitations, the study also benefited from important strengths, including its ecologically valid assessment of daily worries and longitudinal prospective high‐risk design. Findings highlight the need for researchers, policymakers, schools, and families to pay greater attention to the role that school worries may play in the current youth mental health epidemic.

## AUTHOR CONTRIBUTIONS

Conceptualization (Jennifer S. Silk, Samantha R. Silk, Alexandra F. Petryczenko, Cecile D. Ladouceur); Data curation (Kirsten P. McKone, Alexandra F. Petryczenko); Investigation (Jennifer S. Silk, Cecile D. Ladouceur); Formal analysis (Jennifer S. Silk, Kirsten P. McKone, Samantha R. Silk, Alexandra F. Petryczenko); Supervision (Jennifer S. Silk, Cecile D. Ladouceur); Funding acquisition (Jennifer S. Silk, Cecile D. Ladouceur); Project administration (Jennifer S. Silk, Cecile D. Ladouceur, Kirsten P. McKone, Alexandra F. Petryczenko); Resources (Jennifer S. Silk, Cecile D. Ladouceur); Writing – original draft (Jennifer S. Silk, Samantha R. Silk); Writing – review and editing (Kirsten P. McKone, Samantha R. Silk, Alexandra F. Petryczenko, Cecile D. Ladouceur).

## FUNDING INFORMATION

This work was supported by the NIMH under grant R01 MH103241 awarded to J.S.S and CDL.

## CONFLICT OF INTEREST STATEMENT

The authors declare they have no competing or potential conflicts of interest.

## ETHICAL APPROVAL STATEMENT

The study was approved by the University of Pittsburgh Human Research Protection Office on 3/24/2015 (STUDY19070027).

## PATIENT CONSENT STATEMENT

Parent/guardian consent and youth assent were obtained.

## Data Availability

Data are available for the subset of participants who consented to data sharing from the corresponding author upon reasonable request. The data are not publicly available due to privacy or ethical restrictions.

## Footnotes

^1^
School worries were reported less frequently on weekends compared to weekdays (OR = 0.24, *p* < .001).
